# Perspectives on Developing Burn Resistant Titanium Based Coatings—An Opportunity for Cold Spraying

**DOI:** 10.3390/ma16196495

**Published:** 2023-09-29

**Authors:** Sihan Liang, Junlei Tang, Yingying Wang, Tigang Duan, Bernard Normand, Tongzhou Chen

**Affiliations:** 1 Key Laboratory of Optoelectronic Chemical Materials and Devices, Ministry of Education, Jianghan University, Wuhan 430030, China; sihanlia.chim@foxmail.com; 2 School of Chemistry and Chemical Engineering, Southwest Petroleum University, Chengdu 610500, China; tangjunlei@126.com; 3 State Key Laboratory for Marine Corrosion and Protection, Luoyang Ship Material Research Institute (LSMRI), Qingdao 266237, China; duantigang@sunrui.net; 4 INSA Lyon, Université de Lyon, MATEIS UMR CNRS 5510, Bat L. de Vinci, 21 Avenue Jean Capelle, 69621 Villeurbanne, France; bernard.normand@insa-lyon.fr; 5 Wuhan Research Institute of Materials Protection, Wuhan 430030, China

**Keywords:** burn resistant coating, titanium alloy coating, cold spraying

## Abstract

Titanium alloys are crucial lightweight materials; however, they are susceptible to spontaneous combustion under high-temperature and high-pressure conditions, limiting their widespread use in aerospace engines. Improving the burn resistance of Ti alloys is essential for the structural safety and lightweight of aerospace equipment. Burn-resistant Ti alloys, such as Ti-V-Cr and Ti-Cu, however, face limitations such as high cost and low specific strength. Surface coatings provide a cost-effective solution while maintaining the high specific strength and good processability of the base material. Conventional surface treatments, such as laser cladding, result in defects and deformation of thin-walled parts. Cold spray technology offers a promising solution, as it uses kinetic energy to deposit coatings at low temperatures, avoiding defects and deformation. In this paper, we review the current research on burn-resistant surface technologies of Ti alloys and propose a new method of bimetallic coating by cold spraying and low-temperature heat treatment, which has the potential to solve the problem of spontaneous combustion of aerospace engine parts. The strategy presented can also guide the development of high-performance intermetallic compound-strengthened metal matrix composite coatings.

## 1. Introduction

Ti alloys are widely used in engine components such as rotor blades and casings due to their superior performance. However, under certain conditions of temperature, pressure, and airflow speed, Ti alloys can suffer from high-temperature friction and spontaneous combustion, known as “titanium fire” [1]. Currently, the hottest components of turbine engine compressors are made of Ni-based alloys, which are nearly twice as dense as Ti alloys [1,2]. This creates problems related to thermal expansion and connection technology, hindering engine lightweight. To meet the demand for advanced, high thrust-to-weight engines, compressors made of Ti alloys hold great promise for future development. Improving the burn resistance of Ti alloy components is crucial for their wider application and the structural safety and lightweight of aerospace equipment. Lei et al. [3]. conducted a comprehensive exploration of the combustion characteristics and mechanism underlying “titanium fires” originating from aircraft engines, providing detailed insights into existing methods for evaluating fire resistance. Titanium alloys, due to their heightened reactivity, low thermal coefficient, and substantial combustion heat, exhibit susceptibility to ignition. Researchers across the world have attempted to address the problem of Ti alloy spontaneous combustion by developing burn-resistant alloys such as Ti-V-Cr and Ti-Cu. However, the addition of heavy elements such as V, Cr, and Cu results in a decrease in specific strength, complex manufacturing process, high production cost, and limited practical applications. Ti alloys used in aerospace require high plasticity and toughness, making it difficult to find a suitable burn-resistant alloy. The preparation of a burn-resistant coating is an ideal solution to address the “titanium fire” problem as it reduces production costs while maintaining the high specific strength, plasticity, and toughness of the Ti alloy base material. This paper is based on extensive research on fire retardant coatings for aero-engines. Existing and state-of-the-art titanium-based fire retardant coatings are analyzed and discussed in depth. Common problems with existing titanium-based fire retardant coatings are identified, including high cost, low specific strength, and limited high-temperature resistance. We propose to develop titanium-based fire-resistant coatings for use in aerospace engineering, taking advantage of cold spray technology.

## 2. Burn Resistant Ti Alloys

Burn-resistant Ti alloys have become a focus of research in the aerospace industry due to their susceptibility to spontaneous combustion. Currently, the main burn-resistant Ti alloys are Ti-Cr-V and Ti-Cu alloys, which offer flame resistance through (1) creating a protective layer of compact oxides to prevent oxygen penetration, (2) decreasing the adiabatic flame temperature, thus lowering the heat of combustion, (3) enhancing thermal conductivity for faster dispersal of ignition heat, and (4) reducing friction. Ti-V-Cr alloys are represented using β-Ti alloys Alloy C (Ti-35V-15Cr) [4] and Ti40 (Ti-25V-15Cr-0.2Si) [5]. V and Cr increase the cost of Ti-V-Cr alloy, reduce the specific strength, and increase the difficulty of smelting and hot working [2]. Under high temperatures and long-term working conditions, V and Cr cannot stabilize the β phase and prevent the precipitation of the α phase, thereby making its thermal stability difficult to meet the needs of wider applications. Moreover, the flame spreads rather fast as soon as Ti alloys are ignited. This is mainly due to the fact that the combustion heat is high and the thermal conductivity is poor. On the other hand, the peritectic reaction occurs at the interface between the α phase and the β phase at high temperature, and the liquid phase migrates from the phase interface to the inner grains of the β phase [6]. Hence, monophasic α-Ti or β-Ti tissue is more beneficial to high temperature and burn-resistant performance [1,7,8]. α-Ti has high atomic stacking density, slow atomic diffusion, and better thermal stability and creep resistance than β-Ti. Therefore, the evolution from α + β Ti alloys to α-type Ti alloys is an important development trend of Ti alloys used in aero engines in recent years [1,9]. To overcome these challenges, research has shifted towards economical Ti-Cu alloys such as BTT-1 (Ti-13Cu-4Al-4Mo-2Zr), BTT-3 (Ti-18Cu-2Al-2Mo) [10], and Ti14 (Ti-13Cu-1Al-0.2Si) [11], where the presence of Ti_2_Cu in the α-Ti phase greatly improves the burn resistance. The equilibrium phase composition of Ti-Cu burn-resistant alloy is α-Ti + Ti_2_Cu [8]. Ti_2_Cu plays an important role in its burn-resistant performance [10,11,12]. As its melting point is about 990 °C, which is much lower than the ignition temperature of Ti (about 1627 °C in the air), Ti_2_Cu exists in the liquid phase during the combustion process, and its melting process takes away part of the heat and delays the ignition of Ti alloys. Further, the molten Ti_2_Cu formed in the combustion zone transforms the dry friction of the surface into wet friction lubricated by the liquid phase, resulting in a sharp decrease in the frictional heat. Furthermore, the liquid-phase Ti_2_Cu has low oxygen solubility, which hinders the diffusion of oxygen. The addition of Cu also improves thermal conductivity and avoids excessive local temperature, thereby inhibiting combustion.

## 3. Burn Resistant Surface Technologies of Ti Alloys

**Figure 1:**
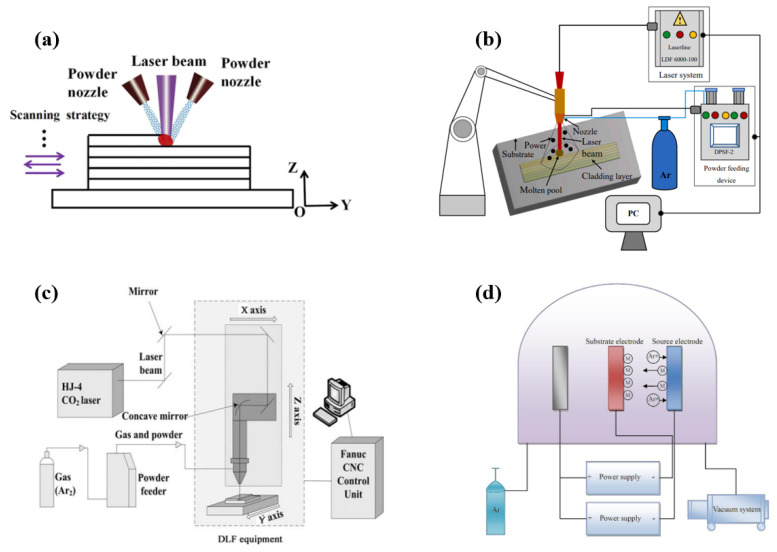
Principles of various laser and double glow plasma surface technologies. (**a**) Laser Solid Form-ing (LSF) [22], (**b**) Laser Cladded (LC) [23], (**c**) Direct Laser Fabrication (DLF) [24], (**d**) Double glow plasma surface metallurgy (DG) [25].

The development of burn-resistant surface technologies for Ti alloys attracts more attention, as the overall burn-resistant alloys tend to be expensive, have low specific strength, complex production processes, and limited practical applications. Recognizing that combustion always originates from the surface, various surface technologies have been proposed to improve the burn-resistance of Ti alloys. These technologies include coatings based on α-Ti and β-Ti alloys and use carriers such as laser beams (e.g., laser cladding), ion beams (ion implantation, double-glow plasma surface alloying, magnetron sputtering, plasma spray), and electron beams (electron beam cladding). Other techniques include electroplating, high-energy mechanical alloying, friction stir machining, and similar methods. Laser cladding [13,14], laser solid forming [15,16], and direct laser fabrication [17,18,19,20,21] were used to prepare burn-resistant coating on the surface of Ti alloys. The related deposition principle is similar (Figure 1), which implies that the surface of the base material is irradiated using different fillers in order to make the deposit and the thin layer of the base material melt simultaneously. Further, after rapid solidification, a deposited layer that is metallurgically combined with the base material is formed. The Ti-based burn-resistant coating prepared using the laser technology is mostly burn resistant β-Ti-V-Cr alloys, which include Ti-25V-15Cr [16,17], Ti-25V-15Cr-0.2Si [14], Ti-35V-15Cr [15], and Ti-25V-15Cr-2Al-0.2C coatings [18,19,20,21].

**Figure 2:**
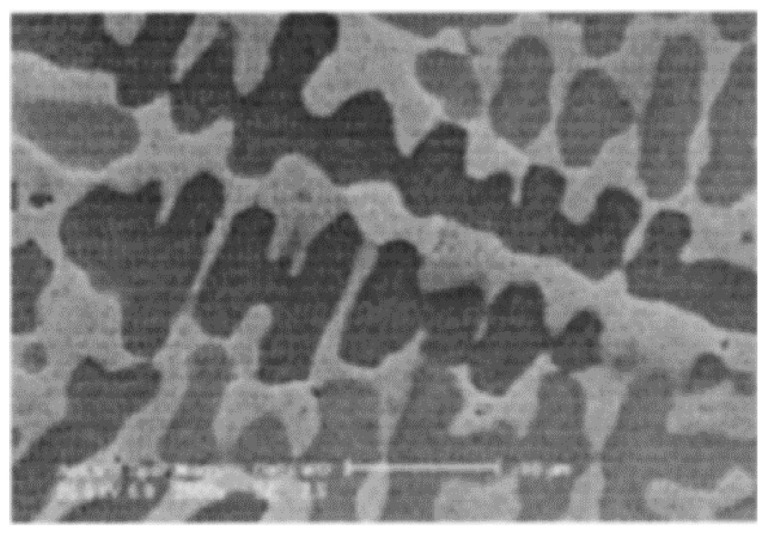
Dendrites observed in Ti-25V-15Cr-2Al-0.2C burn-resistant coating produced by direct laser fabrication [19].

**Figure 3:**
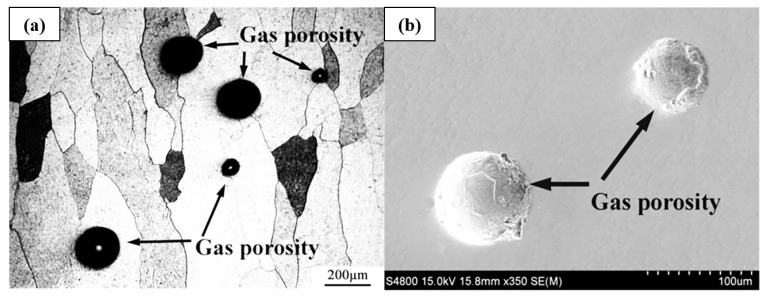
Gas porosity in Ti-25V-15Cr coating by laser solid forming. (**a**) Optical micrograph and (**b**) SEM micrograph [16].

**Figure 4:**
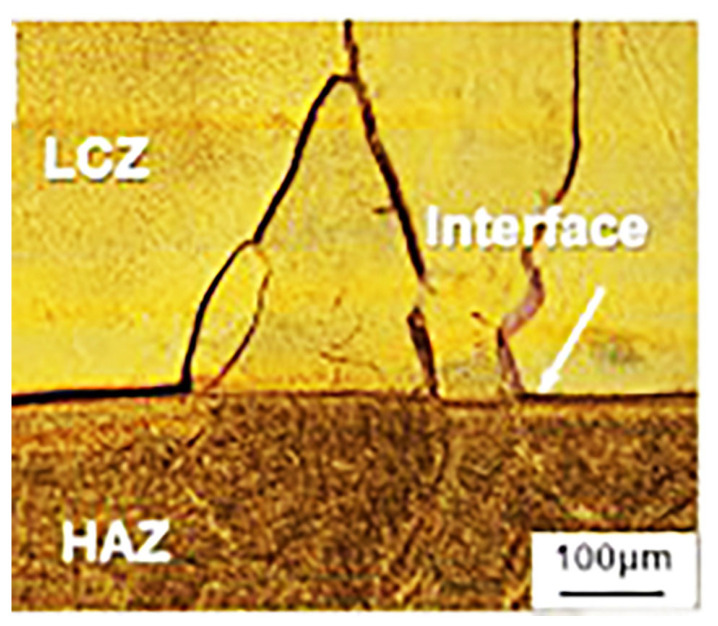
Cracks in Ti-25V-15Cr-0.2Si coating by laser cladding [14].

In laser surface technology, the raw material undergoes melting and then re-solidification. The thermal stress caused due to the rapid cooling rate is unavoidable, and post-treatment is required to eliminate the stress [18]. Moreover, the non-equilibrium solidification process also leads to dendrite (Figure 2) [13,16,19] and defects such as pores (Figure 3) [18] and cracks (Figure 4) [14,15]. Hot isostatic pressing technology is used to improve the coating density [18]. Furthermore, Ti alloys are highly susceptible to oxidation. Wu et al. studied the effect of oxygen content on the Ti-25V-15Cr-2Al-0.2C coating structure deposited using a laser source. The results demonstrated that α-phase precipitation and Ti_2_C dendrite appeared in air deposition, while β-Ti and dispersed Ti_2_C could be obtained in the Ar atmosphere [19]. Therefore, the deposition coating on the surface of Ti alloys using laser technology or using Ti alloys as the deposition raw material should be carried out under a protective atmosphere. In each of the above studies, the vacuum process chamber was filled with high-purity Ar during coating deposition in order to ensure low oxygen content (<5 ppm [18,19,20], <100 ppm [13,14,15,16]).

Laser surface technology also deposits composite coatings such as Ti-Cu-NiCoCrAlTaY composite coating. The Ti-Cu eutectic transition zone formed near the base material delays the combustion, and the oxidation resistance of the outer layer of NiCoCrAlTaY helps to improve flame retardant performance [13]. NiCoCrAlTaY is a kind of MCrAlY coating, and a large number of studies showed that MCrAlY coating has excellent oxidation resistance as an adhesive layer in a thermal barrier coating system [26]. The main preparation parameters of titanium-based flame-resistant coatings prepared by high-energy laser technology are shown in Table 1 below.

NiCr/YSZ double-layer coating also uses a thermal barrier coating system to improve the surface flame performance of Ti alloys, where the NiCr alloy layer that was prepared using the double-glow plasma surface alloying technology is used as the bonding layer. Further, the YSZ ceramic layer that was prepared using multi-arc ion plating technology acts as the blocking layer. The double-layer coating hardness and the burn-resistant performance were found to be superior to Ti-based materials [27]. The Double-glow plasma surface alloying technology, which is also known as the Xu-Tec process, is a surface alloying technology based on ion nitriding technology that can realize metal elements on the surface of the metal materials [28]. This technology utilizes the plasma generated using double-glow photo discharge in a vacuum chamber to provide metal ions and forms a coating on the workpiece surface under the action of thermal diffusion and ion bombardment. Ti-Cu [29,30,31], Ti-Cr [32,33,34], and Ti-Mo [32,35,36] burn-resistant coatings were prepared using this technique. The main parameters of the preparation of titanium-based flame-resistant coatings by the double glow plasma surface alloying technology mentioned in the paper are shown in Table 2 below.

In this methodology, a gradient distribution of Cu, Cr, and Mo is achieved within the coating. It has been observed that the content gradually diminishes with increasing depth, resulting in the absence of a discernible interface between the coating and the substrate. This characteristic significantly mitigates the risk of delamination and peeling. Furthermore, it has been experimentally ascertained that when the Cu content exceeds 12 wt.%, the Cr content surpasses 14 wt.%, and the Mo content exceeds 10 wt.% within the alloy layer, the propensity of the Ti alloy base material to ignite and burn is effectively suppressed [32].

A comparative investigation involving Cu-containing burn-resistant coatings prepared via ion implantation, magnetron sputtering, and double-glow plasma surface alloying has revealed distinctive attributes. Specifically, the ion implantation technique yields a Cu alloy layer with a thickness of less than 0.4 μm, making it challenging to meet practical application requirements. Conversely, magnetron sputtering generates an alloy layer containing Ti_2_Cu and a dual-layer structure comprising a pure Cu film, thereby reducing the overall combustion area. However, this alloy layer is predominantly concentrated within a depth range of 0.2~0.8 μm beneath the surface, rendering it impractical for certain applications. In contrast, plasma surface alloying technology results in a substantially thicker alloy layer, with dimensions such as 200 μm for Ti-Cu, 40 μm for Ti-Cr, and 30 μm for Ti-Mo [30]. Achieving such enhanced thickness necessitates prolonged exposure to elevated temperatures, with optimized infiltration temperatures for Cu, Cr, and Mo measured at approximately 900 °C, 900 °C, and 950 °C, respectively [32].

It is noteworthy that the distance between the source and working electrodes can impact the thickness of the alloy layer. Consequently, obtaining a uniformly deposited layer on the surfaces of complex-shaped components proves to be challenging when employing the double-glow plasma surface alloying technique.

**Figure 5:**
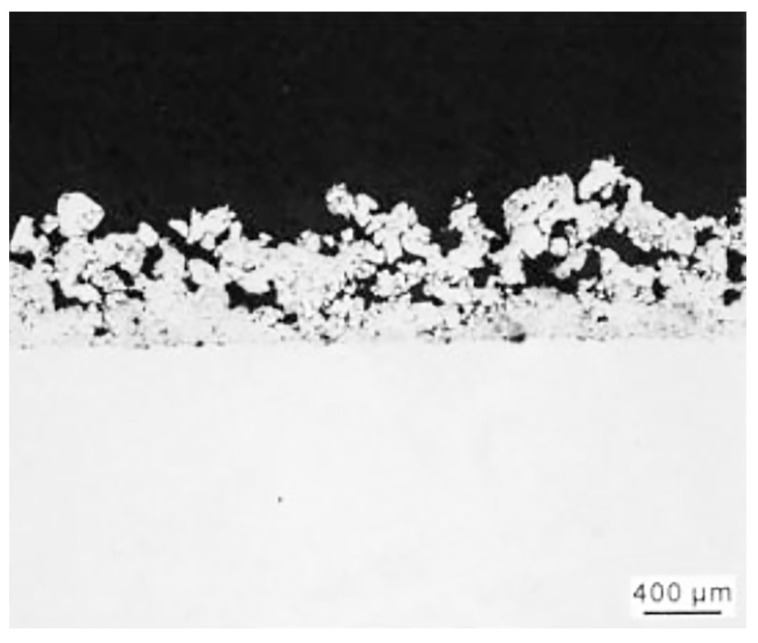
SEM image showing 50% porosity of Ti coating by vacuum plasma spray [37].

Another surface technology that uses plasma is plasma spray, which is a type of thermal spray technology. With a plasma heat source temperature of over 8000 K, this technology has the ability to melt any material. Hence, plasma spray has numerous benefits and is extensively utilized for depositing ceramic coatings, particularly in thermal barrier coatings. Plasma spray is further divided into two categories: plasma spray in the atmosphere and plasma spray under vacuum. The process involves using an arc to melt the raw material, which is then sprayed onto the base material’s surface. However, plasma spray of Ti alloys is known to produce pores and cracks in the coating, with a porosity of up to 50% in vacuum plasma spray (as shown in Figure 5) [37]. Despite this, plasma spray of Ti alloys is often used to prepare porous coatings for applications like artificial tooth roots in the biomedical field and implants like hip and knee joints. The porous surface aids in bone growth, leading to improved implant fixation [38]. However, the pores and cracks can hinder the burn resistance of the coating. For instance, the TiZr-YSZ composite burn-resistant coating produced via a combination of micro-arc pulse ion surface modification and high-energy plasma spray still had visible pores and cracks [39].

In addition to the previously discussed surface technologies, researchers have explored alternative methods, including electron beam cladding and spark deposition technology [40] for the fabrication of Ti-25V-15Cr-0.2Si and TiCrNiVSi0.1 coatings. Similar to laser cladding, electron beam cladding and spark deposition technologies are characterized by high-energy density, which can lead to structural issues such as coarse grains, elevated residual stress, and crack formation. Nevertheless, the application of ultrasonic shock treatment has shown potential in achieving nano-crystallization, thereby enhancing surface-level hardness and burn-resistant properties [41].

High-energy mechanical alloying has also been used to prepare burn-resistant Ti-Cr and Ti-Cu coatings. This method involves modifying a ball mill grinding bottle by cutting a portion of the steel wall and filling it with Ti-6Al-4V base material [42]. However, this method is not suitable for complex parts like blades. Friction stir machining is used to prepare burn-resistant layers composed of β-Ti-rich phase and Ti-Cu intermediate phase, like Ti_2_Cu, but it relies on the rapid rotation and pressure of a stirring head, making it unsuitable for non-planar parts [43].

Electroplating of Cr [44] and Ni [45] can also produce flame-retardant coatings, but electroplating of Ti alloys requires special activation and sensitization treatment. The high current density and long-term electroplating required for large parts have high equipment requirements and also involve the use of acid, alkali, heavy metal solutions, and other raw materials, leading to environmental pollution through waste water, gas, and residue. These methods are gradually being replaced by other surface technologies. The other technical parameters for the preparation of titanium-based refractory coatings mentioned above are shown in Table 3 below.

The mechanical performance of titanium-based fire-resistant coatings is another important criterion for their industrial application. Fire-resistant coatings prepared by various techniques must meet certain mechanical strength and performance requirements, including high hardness, excellent wear resistance, and high bonding strength with the substrate. In addition, fire-resistant coatings must also have matching tensile and shear strength with the substrate material, as well as low residual stress, to ensure their long-term stable operation in the complex environment of high temperature and high pressure in aviation engines. Literature research on coating performance has mostly focused on the influence of coating structure on fire-resistant performance, including the formation of alloying, the depth of element penetration in the coating, and the evolution of metallographic grains in high-temperature environments. The study of the fire-resistant performance of coatings is summarized in Table 4. At present, only a small number of literature has investigated the mechanical properties of flame-retardant coatings, including tensile strength, bonding strength, hardness, and wear resistance, as summarized in Table 5. It is evident that there is a significant lack of in-depth research on the mechanical properties of flame-retardant coatings, highlighting the need for further investigation. Table 6 summarizes the shortcomings of various coating preparation techniques in the preparation of titanium-based refractory coatings. It can be easily found that various high-energy density coating preparation techniques, such as lasers, have problems such as cracks, high residual stresses, and crystal dendrites. In turn, techniques such as electroplating and mechanical alloying have problems such as low coating bond strength, looseness, and limited application range.

In summary, the existing titanium-based flame-resistant coating technologies have not provided an effective solution to the issue of spontaneous combustion in Ti alloy components used in aero engines despite their ability to withstand high temperatures and high energy. It is evident that further advancements are necessary to address this challenge comprehensively.

To overcome the limitations of current surface technologies, there is an urgent need to develop novel methods capable of constructing highly efficient and reliable burn-resistant coatings on Ti alloys. These coatings should effectively minimize the occurrence of defects such as pores and cracks, which can compromise their protective properties. Additionally, it is crucial to minimize the thermal effects on thin-walled components like blades, as excessive heat can lead to structural degradation and reduced performance.

A comprehensive analysis should consider various aspects, including the selection of suitable coating materials and deposition techniques that ensure excellent adhesion and durability. Additionally, the optimization of coating parameters, such as composition, thickness, and microstructure, must be thoroughly investigated to achieve enhanced flame resistance. Advanced characterization techniques should be employed to assess the coating’s mechanical, thermal, and chemical properties, as well as its resistance to oxidation, erosion, and thermal cycling.

Moreover, the development of innovative coating architectures, such as multilayered or gradient structures, may offer enhanced protection against spontaneous combustion. These architectures can effectively reduce the propagation of heat, minimize thermal stresses, and provide improved resistance to aggressive combustion environments.

In conclusion, to address the challenge of spontaneous combustion in Ti alloy components, the development of new methods for constructing highly efficient and reliable burn-resistant coatings is imperative. A comprehensive analysis should consider factors such as material selection, deposition techniques, coating parameters, characterization methods, and innovative coating architectures. By focusing on these aspects, it is possible to minimize defects and thermal effects, ultimately improving the flame resistance of Ti alloy components used in aero engines.

## 4. Cold Spray Technology

The effectiveness of spray technology in the production of metallic coatings has been widely acknowledged. In thermal spray processes, the coating’s quality primarily hinges on the extent of material melting and the velocity of particle deposition. For instance, within the HVOF process, a notable proportion of particles retain their solid state upon impacting the substrate [46,47]. Consequently, achieving a compact and firmly adherent coating through spraying necessitates the provision of both thermal energy and the kinetic energy of particles.

**Figure 6:**
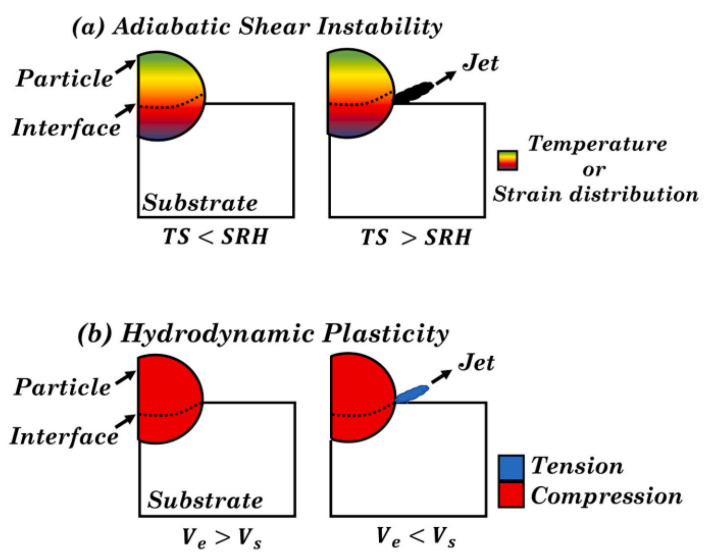
Schematic diagram of cold spraying mechanism [49]. (**a**) Adiabatic Shear Instability, first proposed by Assadi et al. [50], and (**b**) Hydrodynamic Plasticity, proposed by Hassani et al. [51]. TS = thermal softening, SRH = strain rate hardening, Ve = edge velocity, Vs = shock velocity.

In contrast, the cold spray technique utilizes the convergence-divergence effect of a De Laval nozzle to accelerate the particles carried by the gas to very high speeds, ranging from 300 m/s to 1200 m/s [48]. At speeds exceeding the critical velocity, the particles undergo intense plastic deformation and bond with the base material (Figure 6). During the cold spray process, the gas does not undergo combustion, and the high-pressure gas is heated to a lower temperature to increase particle velocity and promote deformation during impact. Throughout the process, the powder raw material remains in a solid state without undergoing the melting and re-solidification metallurgical process characteristic of traditional thermal spray and laser surface technologies.

**Figure 7:**
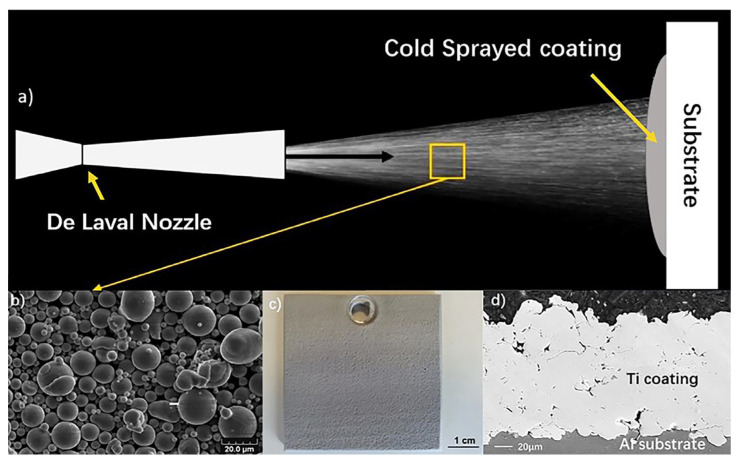
(**a**) cold spray process, (**b**) SEM images of powder raw materials for cold spray, (**c**) pictures of 5 cm × 5 cm cold spray samples, (**d**) SEM of the cross-section of pure Ti coating.

Therefore, the cold spray process is considered an advanced, solid-state coating deposition technology. The technique can be used to deposit a variety of materials, including metals, ceramics, composites, and polymers [52]. Figure 7 illustrates the cold spray process.

The cold spray deposits coatings via a combination of local metallurgical bonding and mechanical interlocking caused by plastic deformation between the particles and at the particle-base material interfaces. This allows for the avoidance of various defects, such as oxidation, grain growth, and phase transformations, which are frequently encountered in high-temperature depositions in both the coating and base material [53]. This makes the cold spray process ideal for depositing heat-sensitive or oxidation-sensitive metal materials, as well as enhancing or repairing such materials. Lightweight alloys (e.g., Ti alloys, Al alloys, Mg alloys), amorphous alloys, and nanocrystalline alloys are susceptible to reactions and phase transitions at high temperatures. Additionally, the plastic deformation of the particles leads to residual compressive stress rather than residual tensile stress in the coating, reducing the risk of cracks and delamination, which are common in thermal spray coating and laser cladding processes. Furthermore, the low residual stress level of the coating makes it possible to prepare ultra-thick coatings without cracking, making cold spray a valuable additive manufacturing technology for creating free-standing metal parts and repairing damaged metal parts [53].

## 5. The Opportunity of Cold Spray for Fabricating Burn-Resistant Ti-Based Coating

Cold-sprayed Ti-alloy coatings exhibit oxygen content similar to that of raw powder materials, effectively preventing titanium oxidation [54]. However, in comparison to face-centered cubic (FCC) phase metals such as Cu and Al, titanium possesses higher yield strength but limited plastic deformation capability, particularly in the case of α-Ti with a hexagonal close-packed (HCP) structure. This leads to incomplete particle deformation and high porosity in cold-sprayed Ti-based coatings, thereby restricting their application. For instance, Figure 7d illustrates the presence of pores in a cold-sprayed pure Ti coating [55].

Khun et al. [55] employed the cold spray technique to produce Ti-6Al-4V (Ti64) coatings with a thickness of 9 mm, which were evaluated in three layers. Their findings revealed that the overall coating exhibited significant porosity and limited corrosion protection for the substrate. Nevertheless, the coating displayed superior hardness and wear resistance compared to the substrate, underscoring the potential for exceptionally strong bonding in cold-sprayed coatings for additive manufacturing of titanium-based refractory coatings for aero-engines.

In contrast, Yin et al. [56] deposited irregular Ti particles onto Al and stainless steel (SS), materials with substantial hardness differences, by adjusting the initial temperature of the powder feeding gas (ranging from 150–600 °C). Their research demonstrated that higher gas temperatures imparted increased kinetic energy and temperature to Ti particles, resulting in more pronounced plastic deformation of the metal and lower porosity. Additionally, the densification of the soft metal matrix was enhanced. However, irregular particles, while possessing higher kinetic energy, also exhibited unfavorable torque for deposition, reducing deposition efficiency. These findings suggest that porosity in the coating can be managed using optimization of cold spraying process parameters.

Hence, two strategies are proposed, which include: (1) to improve the compactness of Ti-based coating, which can be achieved by optimizing spraying process parameters [57], adding a hard reinforcing phase [58], and in-situ shot peening [59]; (2) to use its porous structures to develop functional surfaces, such as artificial tooth roots, implants such as hip and knee joints, etc [60]. As the Cu particles that are completely deformed fill the gaps of Ti particles to obtain a compacted coating structure, the method of the cold spray of Ti-Cu mixed powder is more appropriate for preparing Ti-Cu bimetallic precursor coating. The tight interfacial bonding between the particles is favorable for the interdiffusion of Ti and Cu during the heat treatment. However, the strain rate of the plastic deformation of the particles during the cold spray deposition is as high as 10^8^~10^9^ s^−1^. Further, large strain and high strain rate of the plastic deformation produce supersaturated vacancies and a large number of dislocations in particles, which is conducive to the movement and diffusion of atoms and has an important impact on the evolution of the coating microstructure. For example, the redistribution of Mn and Ni along the grain boundaries was found in the cold spray of FeCoNiCrMn high-entropy alloys [61], which enriches the understanding of the evolution mechanism of the alloy micro-structure under extreme deformation. However, the influence mechanism of the supersaturated vacancies and massive dislocations introduced due to the large strains and high strain rates of plastic deformation in the particles on the diffusion and reaction-diffusion process after the cold spray process is yet to be explored.

**Figure 8:**
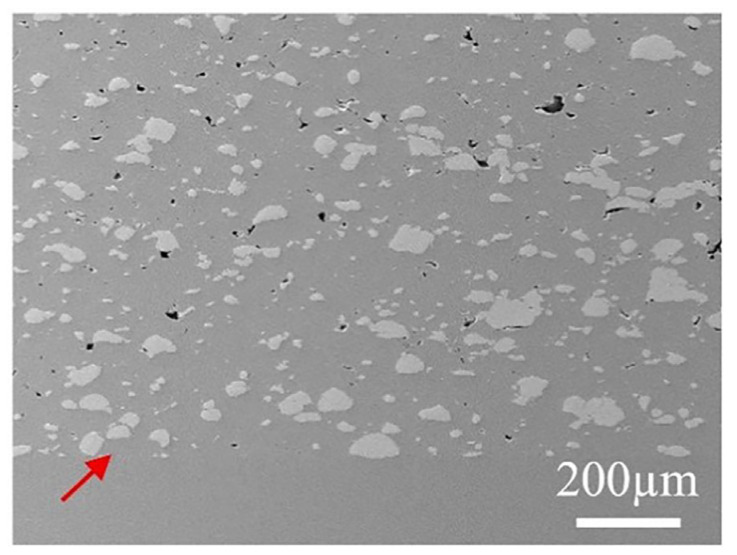
SEM micrographs of cold sprayed Ti6Al4V–CoCr composite coating with pores. The arrows indicate the interface between the coating and substrate [64].

As there is no reaction between different raw materials, the cold spray technology has advantages in composite coating and multi-metal coating deposition. Nevertheless, directly spraying the mixed powder of Ti and Ti_2_Cu to prepare Ti + Ti_2_Cu composite coating is not an ideal method. Initially, adding a hard reinforcing phase reduces the Ti coating porosity; however, it cannot obtain a high-density structure. For instance, Ti-TiC [62], Ti6Al4V-TiC [63], Ti6Al4V-CoCr [64], and other coatings still result in significant pores (Figure 8), especially at the interface between hard particles and Ti. Further, the pores are not conducive to the burn-resistant performance of the coating. Then, Ti_2_Cu is not deformed in cold-sprayed Ti + Ti_2_Cu coating as it mainly relies on the mechanical interlocking effect generated due to the deformation of Ti particles to be imprisoned in the coating. Further, the poorly bonded Ti_2_Cu is easy to fall off, aggravating the friction on Ti. Furthermore, the deposition efficiency of the hard particles in mixed spraying is low, and the content in the coating is significantly lower than that of the original mixed powder composition [63]. The rebound and erosion of the hard particles simultaneously reduce the deposition efficiency of Ti. As a comparison, using the combination of the cold spray technique and the heat treatment in order to prepare α-Ti + Ti_2_Cu coating to obtain better bonding of Ti and Ti_2_Cu, while low-temperature heat treatment reduces the coating brittleness [65] and improve the interface binding of the coating and Ti-based materials [66]. Ti + Ti_2_Cu composite coating is prepared by spraying Ti and Ti_2_Cu mixed powder.

The investigation of producing composite coatings containing intermetallic compounds via low-temperature post-heat treatment of cold-sprayed bimetallic coatings has been explored in various systems, including Al-Ni, Ni-Ti, Fe-Al, and others. Nonetheless, the precise underlying mechanism remains elusive, and the outcomes exhibit inconsistencies.

**Figure 9:**
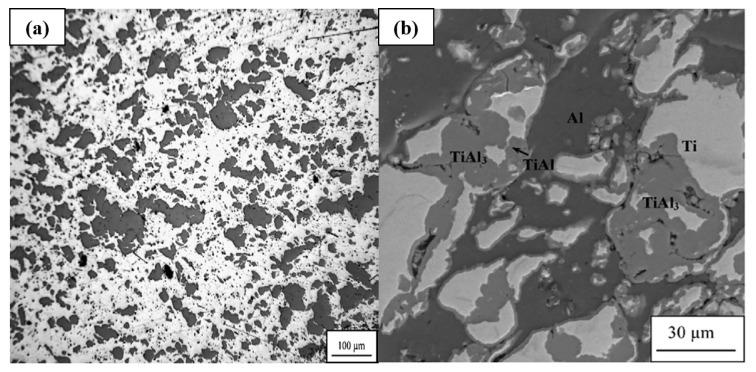
Cold sprayed Ti-Al bimetallic coating by mixture powder, (**a**) as-sprayed and (**b**) after 3 h low-temperature annealing at 650 °C. Both intermetallic phases and pores are obtained after annealing [67].

For example, cold spraying a mixture of Ti and Al powder can yield a dense Ti-Al bimetallic precursor coating devoid of porosity. However, upon heat treatment, the emergence of significant pores is observed, with the formation of these pores exhibiting a positive correlation with temperature and heat treatment duration (see Figure 9) [67]. A TiAl_3_-Al composite coating can be obtained by cold spraying and heat treating a 1:3 mixture of Ti and Al powder, but the porosity increases from 0.17% to 14.69% [68]. In contrast, no discernible pores are evident following the heat treatment of a 1:1 mixture of Ti and Al [69].

**Figure 10:**
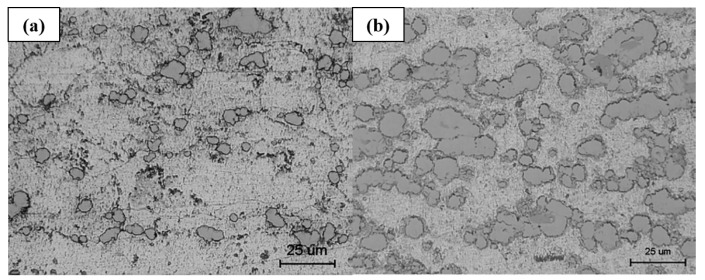
Cold sprayed Ni-Al bimetallic coating by mixture powder, (**a**) as-sprayed, and (**b**) annealing at 450 °C. No porosity increase is observed after annealing [71].

Furthermore, heat treatment of a Ni-Al bimetallic precursor coating may lead to the formation of Ni_3_Al, NiAl, and NiAl_3_, with an augmented increase in porosity observed particularly in cases of low Al content and high heat treatment temperatures [70]. However, heat treatment of Ni-Al bimetallic precursor coating can also result in the formation of NiAl_3_ and Ni_2_Al_3_ without affecting the porosity [71]. Conversely, heat treatment of a Ni-Ti bimetallic precursor coating results in pore formation, but utilizing smaller Ni powder and lower Ti content has been found to prevent their development [72]. Notably, heat treatment of Fe-Al bimetallic precursor coatings does not exhibit any noticeable pores (refer to Figure 10) [73].

These findings underscore the profound influence of heat treatment on the porosity and microstructure of bimetal lic composite coatings. Table 7 illustrates the performance of titanium-based coatings prepared using cold spraying technology and subsequent post-treatment. A more comprehensive examination of the microstructure evolution of cold-sprayed bimetallic precursor coatings during heat treatment is imperative for controlling intermetallic compounds and enhancing the microstructure of composite coatings.

The formation of the pores during the heat treatment is related to the formation conditions of intermetallic compounds, and the mechanism is yet to be further explored. An important reason for the formation of pores is the Kirkendall effect. Due to the difference in the diffusion rate of the materials in which the solid-state phase transition occurs, vacancy easily accumulates on the side of the constituent element with the faster diffusion rate in the diffusion couple. However, the vacancy will be gradually exhausted on the side of the constituent elements with a slower diffusion rate. If the accumulated vacancy cannot be effectively dissolved into the lattice, the supersaturated vacancy will form pores on the side of the constituent elements with a fast diffusion rate. Considering the Ti-Al system as an example, it is observed that the Al-Al metal bond energy is smaller than the Ti-Ti metal bond energy. Therefore, the Al self-diffusion rate is greater than the Ti self-diffusion rate. The results also showed that the Kirkendall voids in the Ti-Al bimetallic precursor coating mainly appear in the Al-rich region. However, due to the lack of research on the diffusion mechanism of Al in Ti, TiAl compounds and the diffusion mechanism of Ti in Al, TiAl compounds, the formation mechanism of Kirkendall voids in cold sprayed bimetallic precursor coating during heat treatment are still unexplored. Meanwhile, the unique interfacial bonding, residual compressive stress, and supersaturated vacancies introduced due to the high strain rate plastic deformation make the nucleation and growth mechanism of Kirkendall voids in solid-state cold sprayed coating different from the microscopic ones in diffusion couples that were prepared using the smelting-welding method. In order to control the void growth that was caused by the Kirkendall effect and regulate the structural evolution of the cold-sprayed coating during heat treatment, the influence of cold-sprayed bimetallic precursor coating micro-structure must be systematically studied. Another source of pores is the density difference between the solid-state phase transition products and reactants. The density of TiAl and TiAl_3_ in Ti-Al alloys is higher than that of Ti, Al mixture, and the transformation of Ti, Al powder into an intermetallic compound phase causes the material to shrink or form pores. The pore formation due to the density difference depends to a large extent on the energy required to form a new interface around the pores. In the compacted cold-sprayed bimetallic precursor coating, the binding force between the particles is strong, and the formation of pores is difficult. Therefore, the volume effect is easy to cause the shrinking of the material. In the case of poor binding, the interface is easily opened and results in pores. Therefore, the contribution of the volume effect caused by the Kirkendall effect and density difference in bimetallic precursor coating to the formation of pores, respectively, is also an issue to be further studied.

The Ti-Cu system shows a contrasting behavior to the Ti-Al system in terms of density, as shown in Table 8. The intermetallic compound Ti_2_Cu formed from the reaction-diffusion of the Ti-Cu bimetallic precursor coating is less dense than the Ti-Cu mixture, which means that the density difference causes the material to expand. This can offset the Kirkendall effect’s contribution to pore formation to some extent. By utilizing the negative density difference between the solid-state phase transition products and reactants in the Ti-Cu system and implementing measures to prevent Kirkendall voids (such as controlling the size of Cu particles, which reduces the diffusion rate), a dense intermetallic compound-enhanced composite coating can be produced from the Ti-Cu bimetallic precursor coating after reaction-diffusion.

A solid-state method that combines cold spray deposition and reaction-diffusion techniques is an effective way to produce burn-resistant coatings on Ti alloys. The low temperature during the cold spray deposition process minimizes thermal impact on the Ti alloy base material and allows for in-situ repair and rapid deposition of ultra-thick coatings with large particles. Mixing Ti and Cu powder and depositing it through cold spray can fill gaps in Ti particles with deformed Cu, leading to a compacted coating structure with reduced porosity. The local metallurgical bonding and mechanical interlocking between Ti and Cu particles promote interdiffusion and affect the volume expansion caused by the density difference between solid-state phase transition products and reactants, thus affecting the growth of Ti_2_Cu. The high strain and strain rate of plastic deformation in cold spray deposition also introduce supersaturated vacancies and numerous dislocations, making the activation energy for solid-state phase transition different from that in a vacancy equilibrium concentration system.

The cold-sprayed particles on the surface contain a higher concentration of defects, such as vacancies and dislocations, compared to their interior. During the low-temperature heat treatment in the reaction-diffusion process, residual compressive stress is released from the coating. The size of the cold-sprayed particles also affects the element diffusion distance and the growth of Kirkendall voids during the reaction-diffusion process. These factors contribute to a unique micro-structure evolution mechanism in the reaction-diffusion process of the cold-sprayed bimetallic precursor coating.

These inherent characteristics of cold-sprayed bimetallic precursor coatings pave the way for future research in this field. Several important aspects remain to be explored. Firstly, there is a need to investigate the mechanisms by which supersaturated vacancies and bulk dislocations, introduced via the large strain and high strain rate during the cold spraying process, affect the diffusion and reaction-diffusion processes. Secondly, the formation mechanism of Kirkendall voids in cold-sprayed bimetallic precursor coatings during heat treatment requires further exploration. Lastly, a deeper understanding of the contributions of the Kirkendall effect and the bulk effect, induced by density differences in the bimetallic precursor coating, to pore formation is necessary.

By addressing these research gaps, we can gain a more comprehensive understanding of the cold spray technology’s potential in the development of flame-resistant Ti-based composite coatings. Through thorough investigation and analysis, we aim to uncover new insights and optimize the performance of these coatings, thereby advancing the field and contributing to enhanced safety in aerospace engine parts.

## 6. Post-Treatment of Cold Sprayed Ti-Based Coatings

As mentioned earlier, the cold spraying technology has significant advantages and broad prospects for the preparation of titanium-based flame retardant coatings for aero-engines. However, the cold-sprayed layer still has problems such as residual stress, porosity, coating bonding strength, wear resistance, and corrosion resistance. It is necessary to eliminate the defects of cold-sprayed coatings and enhance their bonding strength, wear, and corrosion resistance through post-treatment processes.

Post-treatment methods to improve the performance of cold spray coatings mainly include heat treatment and mechanical treatment, of which heat treatment mainly includes laser sintering, annealing treatment, and so on. The existence of microscopic voids in the cold spray coating and the bonding strength with the substrate are the key factors affecting its corrosion and wear resistance [74,75]. T. Marrocco et al. melted the surface of the titanium-based cold spray coating by laser and found that the microscopic voids and defects in the coating were significantly reduced after the laser treatment, which effectively improved the corrosion resistance of the cold spray coating. However, the laser melting treatment of the coating surface will inevitably bring about the problem of high temperature and a negative impact on the coating and the substrate. H. Dong et al. [76] studied the changes in the properties of Ti-6Al-4V coatings after the formation of an oxide layer in the heat treatment, and the study showed that the formation of an oxide layer in the heat treatment would effectively enhance the wear resistance of the coating. Tomila M et al. [77] studied the surface of Ti-Cu coatings by EDM plasma sintering technology and found that the micro voids and defects in the coatings were significantly reduced after the laser treatment, which effectively enhanced the corrosion resistance of cold spray coating technologies and surface treatment of Ti-Cu composite coatings, the study found that the EDM plasma technology effectively eliminated the microcracks present in the coating and significantly improved the hardness of the coating. Ayan Bhowmik et al. [78] through the 600 °C and 950 °C two temperatures of heat treatment, the aero-engine cold spraying titanium-based repair coatings, and found that the heat treatment can effectively eliminate the coating’s residual stresses, eliminate voids in the coating, and enhance the densification. Paloma Sirvent et al. [79] investigated the wear resistance enhancement of Ti-6Al-4V coatings by heat treatment technology and found that the cold-sprayed coatings by heat treatment can effectively prolong the wear life of aero-engines induced by vibration conditions. Felice Rubino et al. [80] studied the effects of laser selective treatment on titanium coatings, and it was found that the oxide layer formed on titanium coatings after laser treatment can maintain the large tempered state of titanium coatings, which leads to a harder oxide layer and enhances the wear resistance of the coatings. A further study by T. Marrocco [81] found that the bonding strength, abrasion resistance, and corrosion resistance of the laser heat-treated improved cold-sprayed coatings, on the other hand, depended on the relevant heat-treatment process’s empirical temperature. Reasonable empirical temperature is the key to ensuring that the mechanical properties of the coating are improved, and the thermal impact of the substrate is minimized.

Mechanical treatment is mainly friction stirring (FSP), which, as a new solid-state processing technology, is a variant of friction stir welding (FSW), which can change the organization and mechanical properties of materials by friction of the heat generated by the material and violent deformation [82]. Therefore, FSP offers a great possibility to eliminate the defects of cold-sprayed coatings. Gang Ji et al. [83] modified cold-sprayed Al coatings on Ti substrates by the FSP technique and found that the hardness and bonding strength of the coating microstructure were enhanced under high rotational speed conditions. F. Khodabakhshi et al. [84] combined cold-spraying and FSP techniques to obtain dense Ti-based coatings and found that a layer with a particle size of less than 1 um was deposited on the surface of the coatings after the FSP technique, and the hardness of the coatings was increased by a factor of nearly 7. C.J. Huang et al. [85] combined the two spraying post-treatments, high-temperature vacuum annealing (HTVA) and stirred friction treatment (FSP), to modify the cold sprayed Ni-Ti coatings. It was found that phase transformation occurs at different HTVA temperatures, leading to the formation of porosity due to the Kirkendall effect. FSP provides for the formation of intermetallic phases in Ni-Ti coatings. Comparison of the microstructure, microhardness, and tribological behavior of the pre-treatment and post-treatment coatings revealed that the microstructure of the modified Ni-Ti coating showed a mechanically alloyed layer with the in-situ synthesized Ni-Ti intermetallic compounds, and no obvious defects were found. The microhardness of the modified coating reached 1003.5 ± 65.9 HV0.1, which was 4.5 times higher than that of the pre-treatment coating (222.5 ± 6.6 HV0.1).

In summary, the heat treatment and friction stirring methods are effective methods applicable to the repair of defects and performance enhancement of cold-sprayed coatings. In the application of cold-sprayed titanium-based flame-resistant coatings, high density, wear resistance, corrosion resistance, high bond strength, and other properties of the flame-resistant coatings can be realized by post-treatment methods to maximize the utility and longevity of cold-sprayed titanium-based flame-resistant coatings in the field of aero-engine applications.

## 7. Conclusions and Outlook

In this study, we have conducted a comprehensive review of recent advances in burn-resistant surface technologies for Ti alloys, aiming to address the critical issue of spontaneous combustion in aerospace engine parts. Our research has led us to propose a novel bimetallic coating approach that combines cold spray deposition with low-temperature heat treatment.

The utilization of cold spray deposition in conjunction with low-temperature heat treatment presents a promising solution to enhance the flame-resistant properties of Ti alloys. Our findings demonstrate the potential of this approach to develop high-performance intermetallic compound-enhanced metal matrix composite coatings. This innovative strategy allows for the formation of a well-bonded coating with superior thermal and mechanical properties, effectively minimizing the risks associated with spontaneous combustion. This approach offers several advantages, including improved adhesion, reduced porosity, enhanced thermal stability, and resistance to aggressive combustion environments. Furthermore, the integration of intermetallic compounds within the coating matrix provides an additional layer of protection against thermal effects and combustion hazards. The development and application of high-performance intermetallic compound-enhanced composites based on the proposed bimetallic coating approach hold great promise in various industries that require materials with excellent flame-resistant properties. These industries include automotive, power generation, and chemical processing, where the prevention of spontaneous combustion is of paramount importance.

It is essential to highlight that further investigation and development of this bimetallic coating approach are warranted to fully unlock its potential. Future research efforts should focus on optimizing the coating parameters, exploring different intermetallic compound combinations, and conducting rigorous performance evaluations under realistic operating conditions. Additionally, the scalability and cost-effectiveness of this approach should be carefully assessed to ensure its practical applicability in industrial settings.

In conclusion, our study provides compelling evidence regarding the significant potential of the proposed bimetallic coating approach in advancing burn-resistant surface technologies for Ti alloys. Through the integration of cold spray deposition and low-temperature heat treatment, we have presented a novel and promising strategy that contributes to the current understanding and development in this field. The findings of this study significantly add to the existing research landscape and offer a viable pathway toward achieving improved performance and enhanced safety in aerospace engine parts.

## Figures and Tables

**Table 1:** Main parameters of high-energy laser alloying preparation technology.

Reference	Powder Flow Rate (g/min)	Linear Velocity (mm/s)	Gas Type	Working Distance (mm)	Laser Power (KW)	Material	
[13]	19.8	132	Ar	15	1.5	Cu	Laser Cladded (LC)
[13]	21	250	Ar	15	2	NiCoCrAlTaY	
[14]	11	10	Ar	7.5	1.8	Ti-6Al-4V	
[15]	10	10	CO_2_	12	2	Ti–35V–15Cr	Laser Solid Forming (LSF)
[16]	4.5	10	CO_2_	12	1.5	Ti-25V-15Cr	
		10			2.1	Ti-25V-15Cr-2Al-0.2C	Direct Laser Fabrication (DLF)
		10			2.4		
		7.5			2.1		
		5			2.1		
		2.5			2.1		
[17]	4	10	CO_2_	12	2.1		
[18]	12	5.83	CO_2_	10	0.75–1.14		
[19]	8~10	2.5–7.5			0.25–0.7		
[20]	9~14	3.3–13.3			0.22–0.51		
[21]	2.64–18.21	1.66			0.755		

**Table 2:** Main parameters of metallurgical alloying preparation process of double glow plasma surface.

	Double Glow Plasma Surface Metallurgy (DG)										
Substrate Material	Ti	Ti-6.5Al-0.3Mo-1.5Zr-0.25Si				Ti-6Al-4V					
Extreme Source Material	Mo	Ti-Mo	Cr	Cr	Mo	Ti-Mo	Cr	Cr	Cu	Cu	Cu
Polar source voltage (V)	860–900	950	950	950	860–900	950	700	950	800	50,000	600–1000
Working gas pressure (Pa)	30	20–50	25	20–50	30	25	25	20–50	25	25	12–36
Permeable layer thickness (um)	50	50	50	50	50	50	60	50	20–250	25–250	210–255
Metal penetration time (h)	3.5	1~6	4	1~6	3.5	4	2	1~6	3.5	0.25–1.41	3
Reference	[36]	[32]	[34]	[32]	[36]	[35]	[33]	[32]	[31]	[30]	[29]

**Table 3:** Main parameters of the alloyed coating by other preparation processes.

Other Process Titanium Based Refractory Coating Parameters		
Plasma Spraying		
Substrate material	TA32	Ti_40_Zr_25_Ni_3_Cu_12_Be_20_
Coating material	ZrO_2_.Y_2_O_3_	
Frequency (Hz)	2000	
Deposition time per unit area (min/cm^2^)	2	
Ar flow (L/min)	20	33
Ar pressure (MPa)		0.7
Electrode speed (r/min)	2600	
Coating thickness (um)	20–40	200–250
Voltage (V)	60	140
References	[34]	
Electron Beam Cladding		
Substrate material	TC4	
Wrapping material	Ti40	
Current (mA)	502	
Welding speed (mm/min)	960	
Welding electron beams (mA)	20	
Frequency (kHz)	20	
References	[35]	
High-Energy Mechanical Alloying		
Substrate material	Ti-6Al-4V	Ti-6Al-4V
Implant material	Cu	Cr, Cu, Ti
Mixing head material	WC-Co	Stainless steel
Stirring head speed (r/min)	350	300–500
Stirring head travel speed (mm/min)	210	
Mixing head shaft shoulder pressure (mm)	0.05	
Forward tilt angle of the mixing head (°)	0	0
References	[36]	[37]
Electrochemical Plating		
Substrate material	TC4	
CrO_3_ concentration (g/L)	500	
Concentrated H_2_SO_4_ concentration (g/L)	2.5	
temperature (°C)	25–30	
Current density (A/dm)	30	
Deposition rate (μm/h)	30	
Plating thickness (um)	15–60	
References	[38]	

**Table 4:** Thickness of titanium-based flame-resistant coatings of different technologies, flame-resistant temperature.

Preparation Technology	Substrate Material	Coating	Coating Thickness (um)	Flame Retardant Performance (°C)	Anti-Oxidation	References
Laser Cladded (LC)	Ti-6Al-4V	Ti-Cu-NiCoCrAlTaY	170	900–1235	better	[13]
Direct Laser Fabrication (DLF)	Ti-6Al-4V	Ti-25V-15Cr-2Al-0.2C	250	500–800	rather poor	[19]
Double glow plasma surface metallurgy (DG)	Ti-6Al-4V	Cu	250	600		[29]
		Cu	250	better		[30]
		Cu	250	better		[31]
		Cr	100	600		[33]
		Cr	100	600–800		[34]
		Ti-Mo	50	800		[35]
	Ti-6.5Al-0.3Mo-1.5Zr-0.25Si	Mo	50	600–800		[36]
High energy mechanical alloying	Ti-6Al-4V	Cu		955–990		[43]
Plasma spraying	TA32	Ti_40_Zr_25_Ni_3_Cu_12_Be_20_		750–900		[39]

**Table 5:** Mechanical properties of titanium-based flame-resistant coatings of different technologies.

Preparation Technology	Substrate Material	Coating	Yield Strength (MPa)	Bond Strength (MPa)	Hardness (HV)	References
Direct Laser Fabrication (DLF)	Ti-6Al-4V	Ti-25V-15Cr-2Al-0.2C	800–900			[18]
			800–1100			[20]
Double glow plasma surface metallurgy (DG)	Ti-6.5Al-0.3Mo-1.5Zr-0.25Si	Mo		35–40	Great	[36]
	Ti	Mo		30–40	Great	[36]
	Ti-6Al-4V	Cu			502	[31]
		Cr			476	[33]
		Ti-Mo			568	[35]
Plasma spraying	TA32	Ti_40_Zr_25_Ni_3_Cu_12_Be_20_	1064	37.6		[39]
	HA	Ti		8		[38]
High-energy electron beam cladding	TC4	Ti40	Lower		531	[41]
**Preparation Technology**	**Substrate Material**	**Implant Materials**	**Porosity**	**Bond Strength (MPa)**	**HSL(GPa)**	**References**
High-energy mechanical alloying	Ti-6Al-4V	Cu	High	High	1.99	[43]
	Ti-6Al-4V	Cr	High	Higher	1.39	

**Table 6:** Defects in Preparation Techniques of Various Refractory Coatings.

Preparation Technology	Coating	Defect	References
Laser Cladded(LC)	Ti-25V-15Cr-0.2Si	High residual stress	[13]
Laser Solid Forming(LSF)	Ti-35V-15Cr	High thermal stress	[14]
	Ti-25V-15Cr	Dendrites, Voids, Thermal stress	[15]
Direct Laser Fabrication (DLF)	Ti-25V-15Cr	Dendrites, Voids, Thermal stress	[16]
	Ti-25V-15Cr-2Al-0.2C	Dendrites, Voids, cracks, High oxygen content, and Significant thermal impact	[17,18,19,20,21]
Double glow plasma surface metallurgy (DG)	Ti-Cu, Ti-Cr, Ti-Mo	Small coating thickness	[27,28,29,30,31,32,33,34,35,36]
Plasma spraying	Ti, TiZr-YSZ	High thermal impact, high porosity, cracks	[37,38,39]
Electron beam cladding	Ti-25V-15Cr-0.2Si	Coarse grains, high residual stress, cracks	[40,41]
High-energy mechanical alloying	Ti-Cr, Ti-Cu	Small scope of application	[42,43]
Plating	Ti-Cr, Ti-Ni	Low bonding strength	[44,45]

**Table 7:** Performance of Titanium-Based Coatings Prepared by Cold Spray Technology and Post Treatment.

Coating Composition	Spray Pressure (MPa)	Spray Temperature (°C)	Porosity (%)	Hardness (HV)	Wear (mm^3^/N⋅m)	References
Ti–6Al–4V	2.8	580	22.4			[54]
Ti–6Al–4V	5	1000	3.5 ± 0.9	458 ± 16	0.52 ± 0.01	[55]
TiAl	2.7	600	0.13			[56]
Ti	2/1	100, 300	2 MPa lower			[57]
Ti-TiAl3	0.5–4	25–600	1–10	850		[58]
Ti6Al4V, Ti	2.8	550	1.3%, 2.7	192/363		[59]
Ti	1.67	260	0.027			[60]
Ti, TiC	4	800		551	42 × 10^−5^	[62]
Ti6Al4V-TiC	4	800	5.0 ± 0.7 (spherical powder) 1.1 ± 0.4 (irregular powder)	218 ± 10 233 ± 8	20 × 10^−5^	[63]
Ti6Al4V–CoCr	4.5	1000	0.9–3.2		2~10 × 10^−4^	[64]
TiAl3-Al	1.8	250	0.17			[68]
Ti, Al	1.5–2	200	5.8–22.8	50–163		[69]

**Table 8:** Ti-Al, Ti-Cu system-related materials crystal density (data source: Materialsproject.org).

Material	Crystal Density (g/cm^3^)	The Density of the Mixture According to the Chemical Formula (g/cm^3^)	Density Change (%)	Solid-State Phase Transition Volume Effect
Ti	4.58	-		-
Al	2.72	-		-
Cu	8.89	-		-
TiAl	3.83	3.65	4.7%	shrink
TiAl_3_	3.40	3.185	6.3%	shrink
TiCu	6.44	6.735	−4.6%	swell
Ti_2_Cu	5.72	6.017	−5.2%	swell

## Data Availability

Data sharing not applicable.

## References

[1] Peters M., Kumpfert J., Ward C.H., Leyens C. (2003). Titanium Alloys for Aerospace Applications. Adv. Eng. Mater..

[2] Zhang S., Zeng W., Zhou D., Lai Y. (2016). Hot Workability of Burn Resistant Ti-35V-15Cr-0.3Si-0.1C Alloy. Mater. Sci. Technol..

[3] Shao L., Li W., Li D., Xie G., Zhang C., Zhang C., Huang J. (2023). A Review on Combustion Behavior and Mechanism of Ti Alloys for Advanced Aero-Engine. J. Alloys Compd..

[4] Zhao Y., Zhou L., Deng J. (1999). Effects of the Alloying Element Cr on the Burning Behavior of Titanium Alloys. J. Alloys Compd..

[5] Zhao Y., Zhu K., Li Y., Liu C., Feng L., Wu H. (2008). One Kind β Type a Low Cost Titanium Alloy.

[6] Shao L., Xie G., Liu X., Wu Y., Yu J., Hao Z., Lu W., Liu X. (2020). Combustion Behaviour and Mechanism of TC4 and TC11 Alloys. Corros. Sci..

[7] Chen Y.N., Huo Y.Z., Song X.D., Bi Z.Z., Gao Y., Zhao Y.Q. (2016). Burn-Resistant Behavior and Mechanism of Ti14 Alloy. Int. J. Miner. Metall. Mater..

[8] Shao L., Xie G., Liu X., Wu Y., Yu J., Feng K., Xue W. (2021). The Effect of Cu Content and Ti2Cu Precipitation on the Combustion Behaviour and Mechanism of Ti-XCu Alloys. Corros. Sci..

[9] Jiang B., Wen D., Wang Q., Che J., Dong C., Liaw P.K., Xu F., Sun L. (2019). Design of Near-α Ti Alloys via a Cluster Formula Approach and Their High-Temperature Oxidation Resistance. J. Mater. Sci. Technol..

[10] Chen Y., Yang W., Bo A., Zhan H., Zhang F., Zhao Y., Zhao Q., Wan M., Gu Y. (2018). Underlying Burning Resistant Mechanisms for Titanium Alloy. Mater. Des..

[11] Zhu K., Zhao Y., Li Y., Liu C., Wu H., Feng L. (2008). One Kind α Type a Low Cost Titanium Alloy.

[12] Yang W., Chen Y., Zhao Q., Zhan H., Xu Y., Zhang F., Zhao Y., Gu Y. (2021). Multiscale Exploit the Role of Copper on the Burn Resistant Behavior of Ti-Cu Alloy. J. Alloys Compd..

[13] Lou L.-Y., Zhang Y., Jia Y.-J., Li Y., Tian H.-F., Cai Y., Lin C.-X. (2020). High Speed Laser Cladded Ti-Cu-NiCoCrAlTaY Burn Resistant Coating and Its Oxidation Behavior. Surf. Coat. Technol..

[14] Zhang F., Qiu Y., Hu T., Clare A.T., Li Y., Zhang L.C. (2020). Microstructures and Mechanical Behavior of Beta-Type Ti-25V-15Cr-0.2Si Titanium Alloy Coating by Laser Cladding. Mater. Sci. Eng. A.

[15] Tan H., Hu T., Wang Y., Zhang F., Qiu Y., Liu T., Fan W., Zhang L.C. (2020). Solidification Effect on the Microstructure and Mechanism of Laser-Solid-Forming-Produced Flame-Resistant Ti–35V–15Cr Alloy. Adv. Eng. Mater..

[16] Zhang F., Liu T., Zhao H., Tan H., Hu G., Zhang Z. (2017). Influence of Processing Parameters on Beta Grain Morphology of Laser Solid Forming of Ti-25V-15Cr Burn-Resistant Titanium Alloy. Int. J. Adv. Manuf. Technol..

[17] Zhang F., Qiu Y., Mei M., Yang X., Hu T. (2018). Burn-Resistant Property of Laser Solid Forming Ti-25V-15Cr Alloy. Rare Met. Mater. Eng..

[18] Wang F. (2012). Direct Laser Fabrication of Ti-25V-15Cr-2Al-0.2C (Wt Pct) Burn-Resistant Titanium Alloy. Met. Mater. Trans. A Phys. Met. Mater. Sci..

[19] Wu X., Sharman R., Mei J., Voice W. (2002). Direct Laser Fabrication and Microstructure of a Burn-Resistant Ti Alloy. Mater. Des..

[20] Wu X., Sharman R., Mei J., Voice W. (2004). Microstructure and Properties of a Laser Fabricated Burn-Resistant Ti Alloy. Mater. Des..

[21] Wang F., Mei J., Wu X. (2006). Microstructure Study of Direct Laser Fabricated Ti Alloys Using Powder and Wire. Appl. Surf. Sci..

[22] Tan H., Wang Y., Wang G., Zhang F., Fan W., Feng Z., Lin X. (2020). Investigation on Microstructure and Properties of Laser Solid Formed Low Expansion Invar 36 Alloy. J. Mater. Res. Technol..

[23] Wu S., Liu Z., Gong Y., Liang X., Wu Y., Zhao X. (2023). Analysis of the Sequentially Coupled Thermal–Mechanical and Cladding Geometry of a Ni60A-25%WC Laser Cladding Composite Coating. Opt. Laser Technol..

[24] Li Y., Bai P., Wang Y., Hu J., Guo Z. (2009). Effect of Ni Contents on the Microstructure and Mechanical Properties of TiC-Ni Cermets Obtained by Direct Laser Fabrication. Int. J. Refract. Met. Hard Mater..

[25] Luo X.X., Yao Z.J., Zhang P.Z., Miao Q., Liang W.P., Wei D.B., Chen Y. (2014). A Study on High Temperature Oxidation Behavior of Double Glow Plasma Surface Metallurgy Fe-Al-Cr Alloyed Layer on Q235 Steel. Appl. Surf. Sci..

[26] Evans H.E., Taylor M.P. (2001). Diffusion Cells and Chemical Failure of MCrAlY Bond Coats in Thermal-Barrier Coating Systems. Oxid. Met..

[27] Wei D.B., Li M.F., Zhou X., Li F.K., Li S.Q., Zhang P.Z. (2021). Preparation of NiCr/YSZ two-layered burn-resistant coating on γ-TiAl alloys based on plasma surface metallurgy and ion plating methods. J. Min. Metall. Sect. B Metall..

[28] Li Z., Zhao W., Ji S., Zhong X., Lian Z. (2020). Research Progress and Trend of Plasma Metallurgy on Titanium Metallic Surface. MATEC Web Conf..

[29] Zhang P.Z., Xu Z., Zhang G.H., He Z.Y. (2005). Preparation of Double Glow Pl as Ma Surface Metall Urgy Treated Ti-Cu Burn-Resistant Alloy. Chin. J. Nonferrous Met..

[30] Zhang G., Zhang P., Huang G., Peng X., Zheng S. (2011). Study on the Burn-Resistant Properties of Titanium Alloy Ti6Al4V Surface by Diffusing Copper. Rare Met. Mater. Eng..

[31] Zhang P.Z., Xu Z., Zhang G.H., He Z.Y., Li Z.Y. (2005). Study of Surface Burn-Resistant Ti-Cu Titanium Alloy. Rare Met. Mater. Eng..

[32] Zhang P.Z., Xu Z., Zhang G.H., He Z.Y., Yao Z.J. (2006). Plasma Surface Alloying of Titanium Alloy for Enhancing Burn-Resistant Property. Trans. Nonferrous Met. Soc. China.

[33] Zhang P.Z., Li Z.Y., He Z.Y., Zhong G.H. (2005). Surface Chromizing of Ti-6Al-4V by Double Glow Plasma Surface Alloying Technology. Ordnance Mater. Sci. Eng..

[34] Zhang P., Xu Z., Zhang G., He Z. (2007). Surface Plasma Chromized Burn-Resistant Titanium Alloy. Surf. Coat. Technol..

[35] Zhang P.Z., Xu Z., Zhang G.H., He Z.Y., Wu H.Y., Yao Z.J. (2007). Surface Plasma Molybdenized Burn-Resistant Titanium Alloy. Key Eng. Mater..

[36] Zang P.Z., Xu Z., Zhang G.H., Zhang Y.M., Wu H.Y., Yao Z.J. (2005). Double Glow Plasma Surface Molybdenizing of Pure Ti and Ti-6Al-4V. J. Nanjing Univ. Aeronaut. Astronaut..

[37] Endres S., Wilke M., Knöll P., Frank H., Kratz M., Wilke A. (2008). Correlation of in Vitro and in Vivo Results of Vacuum Plasma Sprayed Titanium Implants with Different Surface Topography. J. Mater. Sci. Mater. Med..

[38] Liu X., Chu P.K., Ding C. (2004). Surface Modification of Titanium, Titanium Alloys, and Related Materials for Biomedical Applications. Mater. Sci. Eng. R Rep..

[39] Zhang R., Ma X., Yu B. (2019). A Study on Mechanical Properties of Combustion-Resistant and Thermal Barrier Functional Coating Prepared on Titanium Alloy Surface. J. Xiangtan Univ. Nat. Sci. Ed..

[40] Lian Y., Cui M., Han A., Liu Z., Zhang J. (2023). Multi-Criteria Optimization of Automatic Electro-Spark Deposition TiCrNiVSi0.1 Multi-Principal Element Alloy Coating on TC4 Alloy. Coatings.

[41] Zhang Y., Huang C., Liu F., Liu F., Song M., Ke L. (2021). Nanocrystallization of a Ti40 Cladding Layer by Ultrasonic Impact to Improve Burn Resistance. J. Mater. Res. Technol..

[42] Li B., Ding R., Shen Y., Hu Y., Guo Y. (2012). Preparation of Ti-Cr and Ti-Cu Flame-Retardant Coatings on Ti-6Al-4V Using a High-Energy Mechanical Alloying Method: A Preliminary Research. Mater. Des..

[43] Li B., Fu S. (2018). Ti-Cu Flame-Retardant Modified Layer Prepared by Friction Stir Processing on Surface of TC4 Ti Alloy. Chin. J. Nonferrous Met..

[44] Xiong J., Huang J., Xie G., Yu J., Zhang C., Shao L., Wang Y., Li H., He G. (2020). Effect of Electroplating Cr Coating on Combustion Characteristics of TC4 Titanium Alloy. Chin. J. Eng..

[45] Liu H., Chen G., Du L., Lan H., Huang C., Zhang B., Wang H., Zhang W. (2018). Preparation and Characterization of Titanium Alloy Protective Coating. Rare Met. Mater. Eng..

[46] Li C.J., Wang Y.Y. (2002). Effect of Particle State on the Adhesive Strength of HVOF Sprayed Metallic Coating. J. Therm. Spray. Technol..

[47] Chen X., Li C., Li S., Han X., Jiang H., Zhao X. (2023). HVOF Spray Performance Optimization Analysis and Experimental Research of WC–12Co Coating on Ti Alloy. Met. Mater. Int..

[48] Schmidt T., Gärtner F., Assadi H., Kreye H. (2006). Development of a Generalized Parameter Window for Cold Spray Deposition. Acta Mater..

[49] Fardan A., Berndt C.C., Ahmed R. (2021). Numerical Modelling of Particle Impact and Residual Stresses in Cold Sprayed Coatings: A Review. Surf. Coat. Technol..

[50] Assadi H., Gärtner F., Stoltenhoff T., Kreye H. (2003). Bonding Mechanism in Cold Gas Spraying. Acta Mater..

[51] Hassani-Gangaraj M., Veysset D., Champagne V.K., Nelson K.A., Schuh C.A. (2018). Adiabatic Shear Instability Is Not Necessary for Adhesion in Cold Spray. Acta Mater..

[52] Moridi A., Hassani-Gangaraj S.M., Guagliano M., Dao M. (2014). Cold Spray Coating: Review of Material Systems and Future Perspectives. Surf. Eng..

[53] Assadi H., Kreye H., Gärtner F., Klassen T. (2016). Cold Spraying—A Materials Perspective. Acta Mater..

[54] Li W.Y., Zhang C., Wang H.T., Guo X.P., Liao H.L., Li C.J., Coddet C. (2007). Significant Influences of Metal Reactivity and Oxide Films at Particle Surfaces on Coating Microstructure in Cold Spraying. Appl. Surf. Sci..

[55] Khun N.W., Tan A.W.Y., Sun W., Liu E. (2017). Wear and Corrosion Resistance of Thick Ti-6Al-4V Coating Deposited on Ti-6Al-4V Substrate via High-Pressure Cold Spray. J. Therm. Spray. Technol..

[56] Yin S., He P., Liao H., Wang X. (2014). Deposition Features of Ti Coating Using Irregular Powders in Cold Spray. J. Therm. Spray. Technol..

[57] Li C.J., Li W.Y. (2003). Deposition Characteristics of Titanium Coating in Cold Spraying. Surf. Coat. Technol..

[58] Cavaliere P., Silvello A. (2015). Mechanical and Microstructural Behavior of Cold-Sprayed Titanium- and Nickel-Based Coatings. J. Therm. Spray. Technol..

[59] Luo X.T., Wei Y.K., Wang Y., Li C.J. (2015). Microstructure and Mechanical Property of Ti and Ti6Al4V Prepared by an In-Situ Shot Peening Assisted Cold Spraying. Mater. Des..

[60] Cizek J., Kovarik O., Siegl J., Khor K.A., Dlouhy I. (2013). Influence of Plasma and Cold Spray Deposited Ti Layers on High-Cycle Fatigue Properties of Ti6Al4V Substrates. Surf. Coat. Technol..

[61] Yu P., Fan N., Zhang Y., Wang Z., Li W., Lupoi R., Yin S. (2022). Microstructure Evolution and Composition Redistribution of FeCoNiCrMn High Entropy Alloy under Extreme Plastic Deformation. Mater. Res. Lett..

[62] Koricherla M.V., Torgerson T.B., Alidokht S.A., Munagala V.N.V., Chromik R.R., Scharf T.W. (2021). High Temperature Sliding Wear Behavior and Mechanisms of Cold-Sprayed Ti and Ti–TiC Composite Coatings. Wear.

[63] Munagala V.N.V., Chromik R.R. (2021). The Role of Metal Powder Properties on the Tribology of Cold Sprayed Ti6Al4V-TiC Metal Matrix Composites. Surf. Coat. Technol..

[64] Tan A.W.Y., Lek J.Y., Sun W., Bhowmik A., Marinescu I., Buenconsejo P.J., Dong Z., Liu E. (2020). Microstructure, Mechanical and Tribological Properties of Cold Sprayed Ti6Al4V–CoCr Composite Coatings. Compos. B Eng..

[65] Huang R., Sone M., Ma W., Fukanuma H. (2015). The Effects of Heat Treatment on the Mechanical Properties of Cold-Sprayed Coatings. Surf. Coat. Technol..

[66] Blose R.E., Walker B.H., Walker R.M., Froes S.H. (2006). New Opportunities to Use Cold Spray Process for Applying Additive Features to Titanium Alloys. Met. Powder Rep..

[67] Novoselova T., Celotto S., Morgan R., Fox P., O’Neill W. (2007). Formation of TiAl Intermetallics by Heat Treatment of Cold-Sprayed Precursor Deposits. J. Alloys Compd..

[68] Kong L.Y., Shen L., Lu B., Yang R., Cui X.Y., Li T.F., Xiong T.Y. (2010). Preparation of TiAl 3-Al Composite Coating by Cold Spray and Its High Temperature Oxidation Behavior. J. Therm. Spray. Technol..

[69] Novoselova T., Fox P., Morgan R., O’Neill W. (2006). Experimental Study of Titanium/Aluminium Deposits Produced by Cold Gas Dynamic Spray. Surf. Coat. Technol..

[70] Winnicki M., Jasiorski M., Baszczuk A., Korzeniowski M. (2020). Heat-Treatment of Aluminium-Nickel Composite Cold Sprayed Coating. Coatings.

[71] Lee H.Y., Jung S.H., Lee S.Y., Ko K.H. (2007). Alloying of Cold-Sprayed Al-Ni Composite Coatings by Post-Annealing. Appl. Surf. Sci..

[72] Nikbakht R., Assadi H., Jodoin B. (2021). Intermetallic Phase Evolution of Cold-Sprayed Ni-Ti Composite Coatings: Influence of As-Sprayed Chemical Composition. J. Therm. Spray. Technol..

[73] Wang H.T., Li C.J., Yang G.J., Li C.X. (2008). Cold Spraying of Fe/Al Powder Mixture: Coating Characteristics and Influence of Heat Treatment on the Phase Structure. Appl. Surf. Sci..

[74] Hussain T., McCartney D.G., Shipway P.H., Marrocco T. (2011). Corrosion Behavior of Cold Sprayed Titanium Coatings and Free Standing Deposits. J. Therm. Spray Technol..

[75] Marrocco T., Hussain T., McCartney D.G., Shipway P.H. (2011). Corrosion Performance of Laser Posttreated Cold Sprayed Titanium Coatings. J. Therm. Spray. Technol..

[76] Dong H., Bell T. (2000). Enhanced Wear Resistance of Titanium Surfaces by a New Thermal Oxidation Treatment. Wear.

[77] Vidyuk T.M., Dudina D.V., Korchagin M.A., Gavrilov A.I., Bokhonov B.B., Ukhina A.V., Esikov M.A., Shikalov V.S., Kosarev V.F. (2022). Spark Plasma Sintering Treatment of Cold Sprayed Materials for Synthesis and Structural Modification: A Case Study Using TiC-Cu Composites. Mater. Lett. X.

[78] Bhowmik A., Wei-Yee Tan A., Sun W., Wei Z., Marinescu I., Liu E. (2020). On the Heat-Treatment Induced Evolution of Residual Stress and Remarkable Enhancement of Adhesion Strength of Cold Sprayed Ti–6Al–4V Coatings. Results Mater..

[79] Sirvent P., Garrido M.Á., Sharp J., Rainforth W.M., Poza P. (2020). Improving the Oscillating Wear Response of Cold Sprayed Ti-6Al-4V Coatings through a Heat Treatment. Surf. Coat. Technol..

[80] Rubino F., Astarita A., Carlone P., Genna S., Leone C., Memola Capece Minutolo F., Squillace A. (2016). Selective Laser Post-Treatment on Titanium Cold Spray Coatings. Mater. Manuf. Process..

[81] Marrocco T., McCartney D.G., Shipway P.H., Sturgeon A.J. (2006). Production of Titanium Deposits by Cold-Gas Dynamic Spray: Numerical Modeling and Experimental Characterization. J. Therm. Spray. Technol..

[82] Mishra R.S., Ma Z.Y. (2005). Friction Stir Welding and Processing. Mater. Sci. Eng. R Rep..

[83] Ji G., Liu H., Yang G.J., Li C.X., Luo X.T., He G.Y., Zhou L. (2021). Effect of Friction Stir Spot Processing on Microstructure and Mechanical Properties of Cold-Sprayed Al Coating on Ti Substrate. Surf. Coat. Technol..

[84] Khodabakhshi F., Marzbanrad B., Yazdanmehr A., Jahed H., Gerlich A.P. (2019). Tailoring the Residual Stress during Two-Step Cold Gas Spraying and Friction-Stir Surface Integration of Titanium Coating. Surf. Coat. Technol..

[85] Huang C.J., Yan X.C., Li W.Y., Wang W.B., Verdy C., Planche M.P., Liao H.L., Montavon G. (2018). Post-Spray Modification of Cold-Sprayed Ni-Ti Coatings by High-Temperature Vacuum Annealing and Friction Stir Processing. Appl. Surf. Sci..

